# The Intricate Mechanisms of Functional Foods Oyster Mushroom and Fenugreek on Type 2 Diabetic Animal Model

**DOI:** 10.1155/jdr/6209785

**Published:** 2024-12-09

**Authors:** Arafat Hassan Razon, Md. Alauddin, Nisat Farzana, Sanaullah Mazumdar, Md. Ruhul Amin, Md. Mahedi Hassan Tusher, Md. Asrafuzzaman, Nahid Hasan, Mahfuzur Rahman, Muhammad Saiedullah, Begum Rokeya, Md. Omar Faruque

**Affiliations:** ^1^School of Science and Technology, Bangladesh Open University, Gazipur, Bangladesh; ^2^Department of Nutrition and Food Technology, Jashore University of Science and Technology, Jashore, Bangladesh; ^3^Department of Pharmacology, Bangladesh University of Health Sciences, Dhaka, Bangladesh; ^4^Department of Biochemistry and Molecular Biology, Bangladesh University of Health Sciences, Dhaka, Bangladesh

**Keywords:** fenugreek, functional foods, glucose transporter-4, hyperglycemia, oyster mushroom, phospho-AMP-activated protein kinase (p-AMPK), phospho-insulin receptor (p-INSR)

## Abstract

Mushrooms and fenugreek are widely used to reduce hyperglycemia, and fenugreek is also used as a culinary ingredient to enhance flavor and aroma. This study is aimed at investigating the underlying mechanisms of the hypoglycemic effects of mushrooms and fenugreek in a Type 2 diabetic rat model. Adenosine monophosphate (AMP)–activated protein kinase (AMPK) functions to reduce hyperglycemia through insulin-independent pathways and protects beta-cells. Diabetic model rats were administered standard diets supplemented with 5% oyster mushroom powder (mushroom-treated (MT) group) and 5% fenugreek seed powder (fenugreek-treated (FT) group) for 8 weeks. The results showed improvements in both glycemic and lipid profiles, with both oyster mushroom and fenugreek enhancing the phosphorylation of AMPK in muscle tissue. However, no effect on insulin secretion was observed. These findings suggest that both substances reduce hyperglycemia through an insulin-independent pathway. In silico analysis of both mushroom and fenugreek seed extracts revealed bioactive compounds having a strong binding affinity to *α*-glucosidase, which suggests mushroom and fenugreek supplements might control postprandial blood glucose levels.

## 1. Introduction

Diabetes is a significant chronic public health concern on a regional and global scale, affecting both developing and developed countries, and it continues to rank among the leading causes of mortality [1–3]. When left uncontrolled or untreated, diabetes is associated with serious complications, including hypertension, heart failure, kidney failure, anxiety, and certain types of cancer [4–6]. Diabetes is expected to affect 783.2 million people worldwide by 2045, and it has been reported that diabetes has caused an estimated 6.7 million deaths in 2021 [7]. According to this report, 465 million people worldwide, which represents 9.3% of the global population, were affected by diabetes in 2019, and its incidence is rising rapidly [8]. A high-calorie diet and a sedentary lifestyle contribute to the global rise in Type 2 diabetes (T2D) cases [9]. Though the loss of insulin activity and low production of insulin from pancreatic beta-cells are the metabolic markers that characterize T2D, obesity and insulin resistance are the most common causes of T2D [10]. While various medical treatments are now available to manage diabetes, the side effects of synthetic drugs and the high cost of antidiabetic medications can often complicate the effective treatment [11]. Natural products, on the other hand, are alternatives since they are thought to have fewer adverse effects.

The term “functional food” refers to foods or ingredients that offer health benefits beyond basic nutritional value. These foods can help provide additional protection against the development of chronic diseases and conditions. In the case of diabetic patients, various plant-based materials have been used as antidiabetic agents for centuries. Mushrooms and fenugreek are two widely recognized functional foods commonly used in the treatment of diabetes [12]. Fenugreek (*Trigonella foenum graecum*), also known as methi in Bangladesh, and its seeds have long been utilized as a natural remedy for managing diabetes, lowering cholesterol, reducing inflammation, and treating gastrointestinal issues [13]. Research has shown that fenugreek seeds possess hypoglycemic and hypocholesterolemia properties, benefiting both Type 1 and T2D patients, as well as in diabetic rats [14]. Moreover, the oyster mushroom (*Pleurotus ostreatus*) [15] has been consumed for centuries both as food and for medicinal purposes due to their health benefits, particularly in managing diabetes [16, 17] and nutritional qualities, including low energy content, essential fatty acids, and high levels of protein, vitamins, and minerals [18, 19]. Although both mushrooms and fenugreek have shown hypoglycemic effects in animal models, the exact cellular mechanisms underlying these effects remain unclear.

Generally, the insulin and adenosine monophosphate (AMP)–activated protein kinase (AMPK) signaling pathways control glucose and fatty acid metabolism, as well as a variety of aspects of growth, differentiation, and survival. In eukaryotic tissues, both routes are found in abundance [20, 21]. Anabolism is stimulated by the activation of the insulin signaling pathway, whereas AMPK represses anabolism and activates catabolic pathways to conserve ATP. Insulin binds to the insulin receptor (INSR), triggering the insulin signaling cascade by activating the insulin receptor kinase (INSRK) and autophosphorylating the INSR. The activated INSR then interacts with INSR-substrate proteins and phosphorylates them [20, 22]. On the other hand, AMPK, a complex protein molecule found in mammals, is activated by phosphorylation of threonine 172 in response to a range of metabolic stresses [23]. When low levels of ATP are detected, AMPK is activated, resulting in the ratio of AMP to ATP being shifted and altering the cellular redox potential, leading to an increase in the NAD/NADH ratio. AMPK serves as a key regulator, responding to fluctuations in cellular energy levels as well as playing a pivotal role in amendable whole-body metabolism [23, 24]. Besides that, in response of activated AMPK, the glucose transporter-4 (GLUT-4) is intricated in the transfer of extracellular glucose into insulin-sensitive muscle tissues. Furthermore, skeletal muscle tissues are thought to be responsible for up to 80% of glucose transport in the body, and T2D individuals have much lower GLUT-4 expression in both skeletal muscle tissues, indicating that they have a reduced ability to transport glucose [25].

On the other hand, *α*-glucosidase is a key enzyme found in the small intestine that breaks down carbohydrates into glucose by hydrolyzing 1,4-*α*-glucopyranosidic bond [26]. Previous studies reported that the absorption of glucose and the postprandial blood glucose levels could be reduced by the inhibition of *α*-glucosidase activity [27]. Therefore, *α*-glucosidase inhibitors can be effective therapeutic drugs to treat diabetes [28]. *α*-Glucosidase inhibitors such as acarbose, voglibose, and miglitol are the most well-known [29]. However, researchers and consumers are interested in natural sources to mitigate the disease due to some unexpected adverse effects (stomachache, flatulence, and diarrhea) of chemical drugs [30]. Virtual screening (absorption, distribution, metabolism, and excretion (ADME) and molecular docking) is a very effective tool to select the most potent drug hits or leads and trigger drug discovery faster and more efficiently [31].

By considering all of these issues, this study is aimed at investigating whether the reduction of blood glucose levels by mushroom and fenugreek on diabetic model rats uses beta-cell-stimulating insulin-dependent pathway or beta-cell-protecting insulin-independent AMPK activation pathway without stimulating INSR. Additionally, a bioinformatics study was conducted to investigate molecular interaction between phytochemicals (identified in fenugreek and mushroom) and the target proteins.

## 2. Materials and Methods

### 2.1. Place of the Study

The study was conducted in the Nutrition Laboratory, Department of Nutrition and Food Technology, Jashore University of Science and Technology (JUST). Animal facilities were provided by our collaborator, Department of Pharmacology, Bangladesh University of Health Sciences. All animal experiments followed the guidelines outlined in the *Guide for the Care and Use of Laboratory Animals* (8th edition) by the National Research Council (US) Committee [32]. Ethical approval was obtained from the Ethical Review Committee of Bangladesh University of Health Sciences, Dhaka, Bangladesh (BUHS/ERC/EA/23/48/1).

### 2.2. Animals

Long–Evans rats, obtained from the animal house at Bangladesh University of Health Sciences (BUHS) in Dhaka, Bangladesh, were used for this experiment. The animals were maintained under controlled conditions, with a room temperature of 22 ± 2°C, relative humidity of 40%–70%, and a 12-h light–dark cycle.

### 2.3. Development of T2D Model Rats

A single intraperitoneal injection of streptozotocin (STZ) (Sigma-Aldrich, cat S0130) in citrate buffer (pH 4.5), at a dose of 90 mg/kg of body weight, was administered to 48-h-old rat pups (average weight: 7 g) to develop a T2D model [33, 34]. An oral glucose tolerance test (OGTT) was performed to confirm the development of T2D in rats after 3 months of STZ injection. Rats weighing 170%–220 g were fasted overnight for approximately 16 h before receiving an oral glucose load (1 g/kg). Blood samples were collected from the tail vein, and glucose levels were measured at 0 min (before glucose administration) and at 30, 60, and 90 min afterward. Rats with fasting blood glucose levels exceeding 7.0 mmol/L were classified as diabetic models [35] and were subsequently used to investigate the effects of oyster mushroom and fenugreek powder.

### 2.4. Plant Materials and Preparation of Rat Feed

Seeds of the *Trigonella foenum graecum* (fenugreek) plant and oyster mushroom samples were purchased from local producers. Both were botanically authenticated, and voucher specimens (Accession No. 88210 for Trigonella and Accession No. 88211 for oyster mushroom) were deposited at the Bangladesh National Herbarium. *Trigonella foenum graecum* seeds and oyster mushrooms were dried at 40°C for 24 h in a temperature-controlled drier and finely powdered using a powerful mixer grinder; similarly, dried fenugreek seeds were also finely powdered. Rat feed ingredients (flour, wheat bran, maize bran, rice bran, fish meal, beshon, powder milk, salt, oil, vitamins, molasses, and oilcake) were purchased from the local market in Dhaka city. These ingredients were then combined in two separate bowls according to the following proportions: 40% flour, 15% wheat bran, 8% maize bran, 4% rice bran, 10% fish meal, 3% beshon, 4% powder milk, 0.5% salt, 1% oil, 1% vitamins, 0.5% molasses, and 8% oil cake. For the mushroom-treated (MT) group, 5% oyster mushroom powder was incorporated, while the fenugreek-treated (FT) group included 5% fenugreek seed powder. The mixture was blended thoroughly, and water was added to form a dough. This dough was then spread thinly on a baking sheet and dried for 30 min at 150°C.

### 2.5. Experimental Design

A total of 30 rats (*n* = 6 per group) were used in this chronic experimental study over a period of 8 weeks. Nondiabetic control (NDC) group rats and diabetic control (DC) model received normal rat feed (7.5 g food/100 g body weight per day). The glibenclamide-treated (GT) group received glibenclamide at a dosage of 20 mg per kg of body weight, administered orally along with their normal rat feed pellets. The MT group and FT group were each given 5% mushroom powder and crushed fenugreek seeds, respectively, mixed with their normal rat feed pellets. The body weight was recorded weekly. After 8 weeks of supplementation, rats were sacrificed, and muscle tissue was collected. Blood samples were collected and centrifuged at 4000 rpm for 15 min to separate the serum for biochemical analysis. The serum was then stored at −20°C until further examination.

### 2.6. Biochemical Analysis

Blood glucose level was measured by the glucose oxidase–peroxidase enzymatic colorimetric method (Randox Glucose GOD-PAP, GL8318). Serum insulin levels were determined by using the ELISA (enzyme-linked immunosorbent assay) method (Crystal Chem, Cat 90060). Serum total cholesterol, triglycerides, LDL cholesterol, and HDL cholesterol were determined using the enzymatic colorimetric method (Randox Laboratories Ltd., United Kingdom). Antioxidant activity was measured using the DPPH (2,2-diphenyl-1-picrylhydrazyl) method (DPPH from Sigma-Aldrich, D9132).

### 2.7. Reverse-Transcriptase PCR (RT-PCR)

The mRNA was isolated from the muscle tissue using TRIzol reagent (ThermoFisher, TRIzol 15596026) (1 mL/100 mg of tissue). The concentration of RNA in each solution was determined by using a 260 nm absorbance measurement, and the mRNA was transformed into a single strand of complementary DNA (cDNA) by utilizing a high-capacity cDNA reverse transcriptase kit (Promega Corp. United States, Cat. A3500) [36]. In 10 *μ*L reaction volume, 1 *μ*g of mRNA was utilized. To observe the expression of GLUT-4, 3.0 *μ*L of cDNA was utilized for PCR. The following primers were used in this study: Forward: 5⁣′-GGG CTG TGA GTG AGT GCT TTC-3⁣′ and reverse: 5⁣′-CAG CGA GGC AAG GCT AGA-3⁣′. To conduct the PCR for the housekeeping gene GAPDH, the following primers were used: Forward: 5⁣′-TGC TGG GGC TGG CAT TGC TC-3⁣′ and reverse: 5⁣′-TCC TTG CTG GGC TGG GTG GT-3⁣′. HotStar Taq DNA Polymerase (QIAGEN, cat 203203) was used for the PCR reaction. The final product was separated using a 3% agarose gel electrophoresis (agarose from MP Biomedical, cat AGAH0100). By using ImageJ software, the band intensities of the PCR products were assessed, and variations in GLUT-4 expression were standardized to the housekeeping gene GAPDH.

### 2.8. Western Blotting

RIPA lysis buffer (200 mg tissue/1 mL buffer: Thermo Scientific, United States, cat. No. 89900) was used to extract proteins from the muscle tissue. The protein concentration was quantified using a Bradford reagent. To detect specific protein expression, the Western blotting technique was carried out throughout the study as described previously [24]. In this study, sodium dodecyl sulphate polyacrylamide gel electrophoresis (SDS-PAGE) (8% separating and 4% stacking gel) was used to resolve the total cell lysates (acrylamide/bis-acrylamide from Sigma-Aldrich, A7802). Protein was then transferred to a polyvinylidene difluoride (PVDF) (ThermoFisher, cat 22860) membrane in a transfer buffer (Tris base, glycine, 20% methanol). After that, a 5% BSA (bovine serum albumin) (Sigma-Aldrich, cat A2153) solution was used to block the membranes, and these membranes were then incubated with primary rabbit monoclonal antibodies (Thermo Scientific, United States, p-AMPK, Thr 172 polyclonal antibody, cat PA5-17831; p-INSR rabbit monoclonal antibody, cat 701070) (1:100). After incubation with primary antibodies, the membranes were further incubated with a 1:2000 dilution of the secondary horseradish peroxide-anti-rabbit IgG (Thermo Scientific, United States, cat G21234.) for 1 h. Protein bands were detected by enhanced chemiluminescence (ECL) (Thermo Scientific, cat 32132) solution on autoradiography film. We utilized a Thermo solution comprising ECL Solution A and ECL Solution B. A mixture was prepared by combining 4 mL of ECL A with 100 *μ*L of ECL B. Then, membranes were dipped into this mixture for 2 min, the membranes were then placed on the X-ray plate of autoradiography, and the band intensities were determined by ImageJ software. Changes in the p-AMPK and p-INSR levels were normalized to the housekeeping protein *β*-actin (beta-actin polyclonal antibody, Thermo Scientific, cat PA1-46296).

### 2.9. The Histopathological Analysis of Pancreatic Tissue

The pancreas tissue samples collected for histopathological analysis were fixed in a 10% formalin solution for 48 h and subsequently washed under running water for 8 h. The samples were treated with alcohol and xylene before being embedded in paraffin. Thin sections (4 *μ*m) were taken from each block and placed onto slides for histopathological analysis, followed by staining with hematoxylin–eosin (HE). The relevant areas were photographed and analyzed using a light microscope.

### 2.10. Bioactive Compound Identification by Gas Chromatography–Mass Spectrometry (GC-MS)

GC-MS analysis was carried out with Clarus 690 gas chromatograph (PerkinElmer, California, United States) using a column (Elite-35, 30 m length, 0.25 mm diameter, and 0.25 *μ*m thickness of film), and it was equipped with Clarus SQ 8 C mass spectrophotometer (PerkinElmer, CA, United States). One microliter sample was injected (splitless mode), and pure helium (99.999%) was used as a carrier gas at a constant flow rate (1 mL/min) of 40 min run time. The sample was analyzed in EI (electron ionization) mode at high energy (70 eV). Though inlet temperature was constant at 280°C, column oven temperature was set at 60°C (for 0 min), raised at 5°C per minute to 240°C and hold for 4 min [37]. The sample compounds were identified and compared to the National Institute of Standards and Technology (NIST) database.

### 2.11. In Silico Study

#### 2.11.1. Building a Chemical Library and ADME Profiling

A chemical library was constructed from 83 compounds that were found via GC-MS analysis. The ADMEs of these compounds were evaluated by their SMILES string using the SwissADME (http://www.swissadme.ch) online server [38]. Finally, seven compounds were isolated and selected for the study depending on their pharmacokinetic properties and Lipinski's rule. Acarbose was considered as control drug against the target protein.

#### 2.11.2. Preparation of Ligands

The 3D structures of the selected GC-MS compound and the control drug were downloaded from the PubChem database (https://pubchem. http://ncbi.nlm.nih.gov/) in the SDF format. Energy minimization of the ligands was carried out by enforcing the UFF and conjugate gradients algorithm of PyRx [39].

#### 2.11.3. Preparation of the Targeted Protein

The Protein Data Bank (PDB) structure of the human *α*-glucosidase enzyme (PDB ID: 5NN8) was downloaded from the RCSB PDB. The retrieved protein structure was finalized for further procedures using the Discovery Studio 2021 (https://www.3ds.com/products-services/biovia/products/molecular-modeling-simulation/biovia-discovery-studio/) by removing the cofactors, water molecules, and metal ions attached to the protein. Energy minimization is required to stabilize the protein structure, which was ensured by utilizing the GROMOS96 force field of SWISS PDB Viewer (https://spdbv.unil.ch/) [40].

#### 2.11.4. Molecular Docking Study

In structural biology, molecular docking is renowned as a reliable technique, especially in CADD processes [41]. PyRx is an open-access virtual screening tool dedicated to the screening of a vast range of libraries of compounds against a particular drug target. To figure out the best binding interaction of our desired protein and the hit compounds, we used AutoDock Vina wizard of the PyRx tool for molecular docking [39]. All the default configurations and parameters of PyRx were retained during the docking process based on the highest binding energy (kilocalories per mole) for evaluation. A binding site was created by the digital removal of acarbose with the radius of 25 Å and coordinates *x* = −16.250, *y* = −30.868, *z* = 94.530. We used the BIOVIA Discovery Studio 2021 visualizer to observe the binding pose and nonbond interaction of the protein–ligand complex.

### 2.12. Statistical Analysis

The Statistical Package for Social Science (SPSS) software Windows Version 20 (SPSS Inc., Chicago, Illinois, United States) was used for statistical analysis. The data were reported as the mean ± SD. ANOVA (Bonferonni) test was used for the comparison of fasting blood glucose among the five different groups. Student's “*t*” test was used for the comparison of body weight of treated rats with diabetic model rats, and paired *t*-tests (0 day vs. 56^th^ day within a group) were used to analyze the statistical difference between the groups. A two-tailed *p* value of < 0.05 was considered statistically significant.

## 3. Results

### 3.1. OGTT

Three months after STZ induction, rats were classified as diabetic if their blood glucose levels exceeded 7 mmol/L. The diabetic rats were then grouped with weight-matched controls, and an OGTT was conducted to confirm the presence of T2D.  Table 1 illustrates the changes in serum glucose levels across the various groups of rats. Baseline serum glucose levels were 5.60 ± 0.33, 11.53 ± 0.9, 11.30 ± 0.6, 10.56 ± 1.1, and 10.98 ± 2.1 mmol/L (0 min, mean ± SD) in NDC rats, DC rats, and GT, MT, and FT diabetic model rats, respectively. Fasting blood glucose levels of all the experimental groups were significantly different compared to nondiabetic rats in the ANOVA (Bonferroni) test. After 90 min of glucose administration, the serum glucose level was 8.79 ± 1.06, 18.0 ± 4.79, 19.89 ± 4.96, 16.97 ± 4.32, and 22.97 ± 3.48 in the experimental groups. As expected, serum glucose level increased only by 3 mmol/L in the NDC group. Conversely, all rats subjected to STZ at the neonatal stage exhibited partial destruction of *β*-cells, resulting in their inability to effectively respond to the oral glucose challenge.

### 3.2. Changes in Body Weight

**Figure 1:**
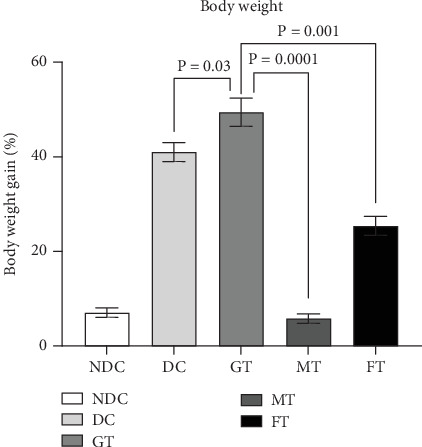
Effect of oyster mushroom and fenugreek on body weight (g) of Type 2 diabetic model rats. NDC, nondiabetic control; DC, diabetic control; GT, glibenclamide treated. Data presented as mean ± standard deviation (*M* ± SD).

Figure 1 illustrates the variation in body weight across different groups of rats. Body weight was recorded on a weekly basis throughout the study. While all five groups exhibited an upward trend in weight gain, the increment was significantly lower in the MT group (5.8%, *p* = 0.0001) and FT group (25%, *p* = 0.001) compared to the DC group (41.0%).

### 3.3. Effect of Mushroom and Fenugreek on Fasting Serum Glucose Level of Diabetic Model Rats

**Figure 2:**
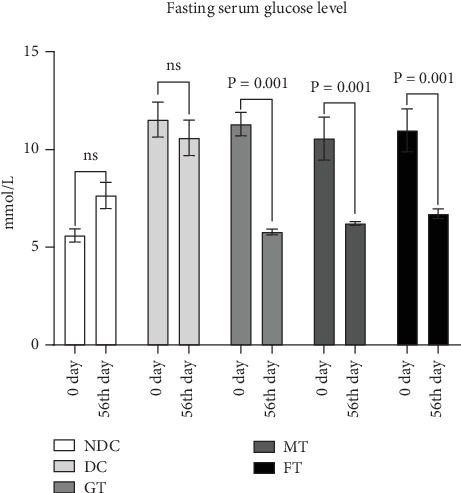
Effect of mushroom and fenugreek on fasting serum glucose level in different groups at 0 day and 56th day of the experiment. NDC, nondiabetic control; DC, diabetic control; GT, glibenclamide treated; ns, not significant. Data presented as mean ± standard deviation (*M* ± SD).

Figure 2 shows that fasting serum glucose levels were 5.60 ± 0.33, 11.53 ± 0.9, 11.30 ± 0.6, 10.56 ± 1.1, and 10.98 ± 2.1 mmol/L on the initial day of the NDC, DC, GT, MT, and FT groups, respectively. After eight consecutive weeks of chronic supplementation with the experimental diets, the fasting serum glucose levels were recorded as 7.65 ± 0.67, 10.6 ± 0.94, 5.78 ± 0.14, 6.22 ± 0.10, and 6.71 ± 0.25 mmol/L in the NDC, DC, GT, MT, and FT groups, respectively. MT and FT groups significantly reduced fasting serum glucose levels like the GT group (standard drug) after 8 weeks compared to baseline (*p* = 0.001), as indicated by a paired *t*-test.

### 3.4. Serum Insulin Level

**Figure 3:**
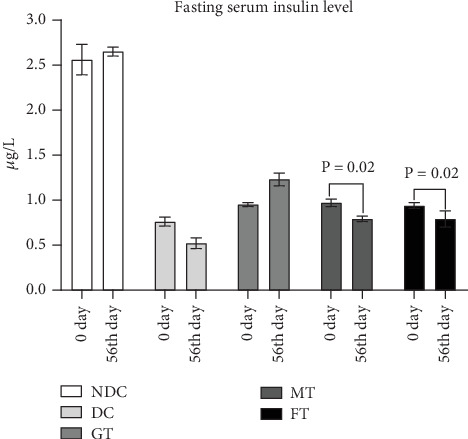
Effect of oyster mushroom and fenugreek on serum insulin level of diabetic model rats. NDC, nondiabetic control; DC, diabetic control; GT, glibenclamide treated. Data presented as mean ± standard deviation (*M* ± SD).

Figure 3 illustrates the impact of mushroom and fenugreek powder supplementation on serum insulin levels in a diabetic rat model. After 8 weeks of chronic supplementation, both MT and FT groups significantly decreased serum insulin levels (*p* = 0.02, paired *t*-test) in STZ-induced diabetic rats compared to baseline levels. In contrast, supplementation with the drug glibenclamide led to an increase in serum insulin levels. We found that fenugreek and mushrooms decreased the insulin level from 14% to 9%, but glibenclamide increased the insulin level by 29% compared to the diabetes control group (data not shown in the figure).

### 3.5. Effect of Mushroom and Fenugreek on Lipid Profiles

Chronic supplementation of oyster mushroom and fenugreek powder significantly modulates the lipid profiles of STZ-induced diabetic rats (Table 2). We found that the MT group significantly decreased serum cholesterol levels. FT diabetic model rats significantly increased cholesterol levels (19%); however, the standard drug GT group showed a decreased level of both triglyceride (3%) and total cholesterol level (13%). Both the mushroom and FT group significantly increased HDL levels (4%–25%) and decreased LDL levels (33%–40%), whereas the DC group increased their lipid profile.

### 3.6. Antioxidant Level

**Figure 4:**
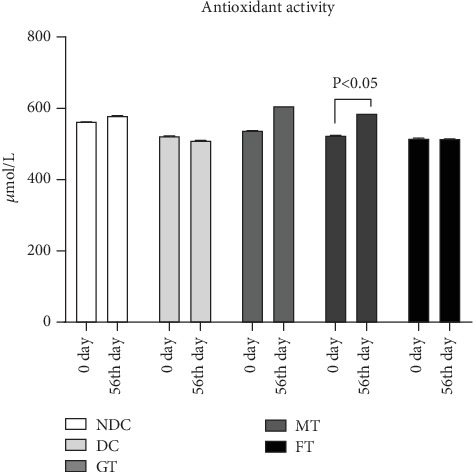
Effect of oyster mushroom and fenugreek on total antioxidant status in diabetic model rats. NDC, nondiabetic control; DC, diabetic control; GT, glibenclamide treated. Data presented as mean ± standard deviation (*M* ± SD).

Figure 4 describes the effect of mushroom and fenugreek supplementation on the antioxidant status of STZ-induced T2D model rats. After 8 weeks of chronic supplementation, the MT group significantly increased antioxidant levels by 11% (*p* < 0.05, paired *t*-test), whereas no significant changes were observed in the FT group. Additionally, rats treated with the drug glibenclamide demonstrated a 12% increase in antioxidant levels.

### 3.7. Effect of Mushroom and Fenugreek on p-AMPK and p-INSR Activation

**Figure 5:**
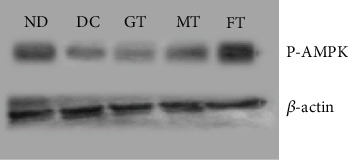
Effect of mushroom and fenugreek on AMPK activation. Lane 1: nondiabetic (ND) rat; Lane 2: Type 2 diabetic control (DC) rat; Lane 3: glibenclamide-treated (GT) Type 2 diabetic rat; Lane 4: mushroom-treated (MT) Type 2 diabetic rat; Lane 5: fenugreek-treated (FT) Type 2 diabetic rat. Three hundred micrograms of protein lysates from rat muscle tissues was separated using 8% polyacrylamide gel, proteins were transferred to the PVDF membrane, after blocking with 1% BSA, membranes were stained with anti-pAMPK antibody, and equal amounts of proteins were also used for a housekeeping protein beta-actin analysis.

**Figure 6:**
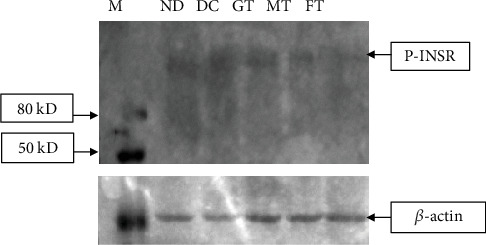
Effect of mushroom and fenugreek on INSR activation. Lane 1: nondiabetic (ND) healthy rat; Lane 2: diabetic control (DC) rat; Lane 3: glibenclamide-treated (GT) diabetic rat; Lane 4: mushroom-treated (MT) diabetic model rat; Lane 5: fenugreek-treated (FT) diabetic model rat. Three hundred micrograms of protein lysates from rat muscle tissues was separated using 8% polyacrylamide gel, proteins were transferred to the PVDF membrane, after blocking with 1% BSA, membranes were stained with anti-p-INSR antibody, and equal amounts of proteins were also used for a housekeeping protein beta-actin analysis.

We found that 8 weeks of chronic supplementation with mushroom and fenugreek powder significantly increased p-AMPK levels (Figure 5), whereas p-INSR levels were decreased in muscle tissue (Figure 6). The Western blot analysis demonstrated a notable increase in the band intensity of p-AMPK in both mushroom and FT diabetic model rats. However, we noticed a significantly lower band intensity of p-AMPK among nondiabetic, diabetic, and GT T2D model rats. The detection of band intensities of p-INSR from muscle tissue was decreased significantly in both mushroom and FT diabetic model rats. On the other hand, we found a significantly higher band intensity of p-INSR among nondiabetic, diabetic, and GT T2D model rats.

### 3.8. Effect of Mushroom and Fenugreek on GLUT-4 mRNA Expression

**Figure 7:**
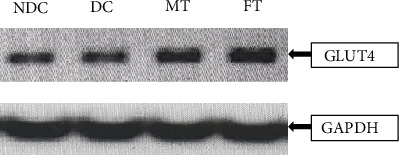
Effect of oyster mushroom powder and fenugreek on the expression of GLUT-4 mRNA in Type 2 diabetic model rats. Lane 1: nondiabetic rat muscle; Lane 2: diabetic control rat; Lane 3: mushroom-treated rat; Lane 4: fenugreek-treated rat. One microgram of mRNA from muscle tissue was used for cDNA preparation, and three microliters of cDNA from each was used for PCR (35 cycles).

After 8 weeks of chronic supplementation with mushroom and fenugreek powder, a significant increase in GLUT-4 levels was observed in muscle tissue (Figure 7). Analysis through mRNA extraction and qRT-PCR revealed a marked elevation in the band intensity of GLUT-4 mRNA in the mushroom and FT T2D model rats compared to the DC group.

### 3.9. Effect of Mushroom and Fenugreek on Pancreatic Tissue

**Figure 8:**
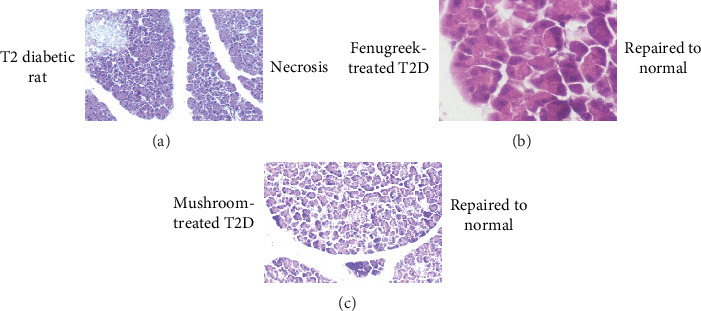
Histopathology of the pancreatic tissue of control and treatment groups of fenugreek- and mushroom powder–induced diet. (a) Diabetic control (STZ-induced diabetic rats), degeneration and necrosis in pancreatic cells; (b) Group II: fenugreek-treated diabetic rats, with improved degeneration and necrosis in islet cells; and (c) Group III: oyster mushroom-treated Type 2 diabetic rats, with improved degeneration and necrosis in islet cells.

The pancreatic histopathology (Figure 8) shows the histopathological structure of the pancreatic tissue. It was observed that the pancreatic *β*-cells of the diabetic rats were completely damaged due to STZ induction. It was revealed that the degeneration and necrosis within the pancreatic islet cells decreased significantly, and the pancreatic structure was restored in the diabetic rats after the fenugreek- and mushroom-containing diet administrations.

### 3.10. Bioactive Chemical Compound Screening by GC-MS

**Figure 9:**
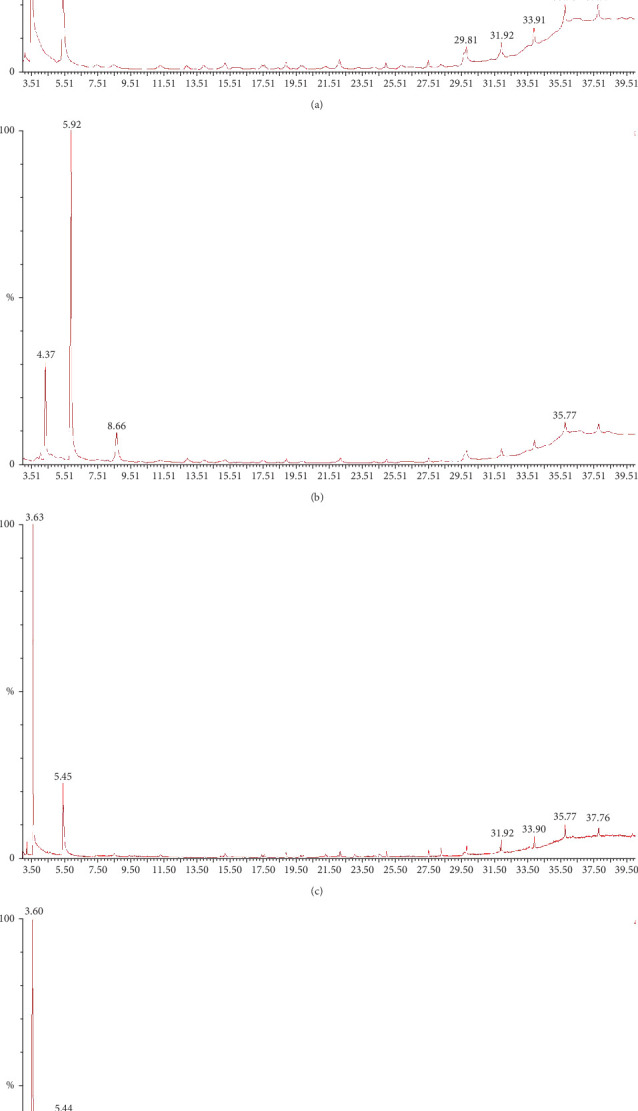
GC-MS chromatogram of phytochemicals. (a) Alcohol-extracted fenugreek, (b) water-extracted fenugreek, (c) alcohol-extracted mushroom, and (d) water-extracted mushroom.

An examination of GC-MS was used to identify distinct chemical substances in the columns of column chromatography. Eighty-three compounds were identified from four samples as ethanol and water extraction of fenugreek and ethanol and water extraction of mushroom. The chromatogram of GC-MS showed the number of compounds and retention time via spectrum (Figure 9). Table S1 shows the phytochemical compositions of different extractions of fenugreek and mushroom with retention time, chemical name, molecular weight, and percent peak area. The compound propane, 2-chloro-2-nitro (C_3_H_6_ClNO_2_) was found mostly (41.38%) in ethanol-extracted fenugreek sample, whereas the compounds oxime-, methoxy-phenyl- (C_8_H_9_NO_2_), 4-ethylbenzoic acid, and 2-methylbutyl ester (C_14_H_20_O_2_) displayed percentage peak area of 15.48% and 5.44%. The resting compound remained below 3%. On the other hand, water extracted fenugreek sample contains the compounds, namely, 4-ethylbenzoic acid, cyclopentyl ester (C_14_H_18_O_2_), 4-chloro-2-isothiocyanato-1-phenoxybenzene (C_13_H_8_ClNOS), C_3_H_6_ClNO_2_, and diazene, 1-(2,4-dinitrophenyl)-2-phenyl (C_12_H_8_N_4_O_4_) as 36.13%, 20.05%, 11.44%, and 9.72%, respectively. Moreover, the compounds C_3_H_6_ClNO_2_ and C_8_H_9_NO_2_ showed 29.13% and 15.93% in ethanol-extracted mushroom samples. Similarly, these two compounds are found mostly also in water-extracted mushroom samples.

### 3.11. Phytochemical Screening by Pharmacokinetics

To investigate the pharmacokinetics properties of the identified phytochemicals ADME testing was done through the SwissADME online server and found better results for six compounds from all of the compounds. In Table 3, the selected six compounds showed good intestinal absorption as well as drug-like characteristics based on Lipinski's rule (molecular mass less than 500 Da, partition coefficient not greater than 5, hydrogen bond donors less than 5, and hydrogen bond acceptors less than 10) of violation. All of the compounds' topological polar surface area (TPSA) was less than 130 Å^2^, and synthetic accessibility was easy. Interestingly, these six compounds showed a higher GI absorption rate than the control drug.

### 3.12. Molecular Docking and Postdocking Analyses

**Figure 10:**
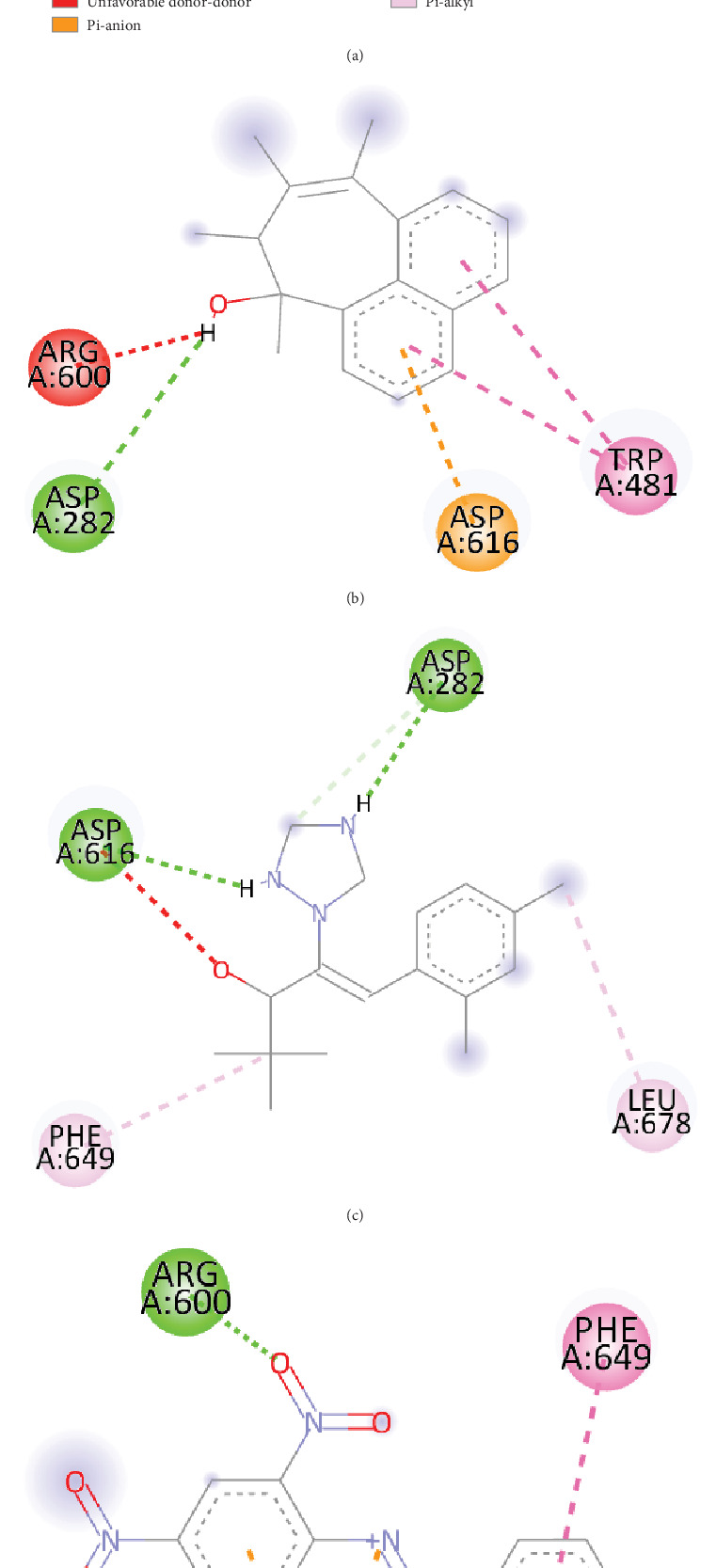
Binding pose and molecular interaction of the fenugreek compounds. (a) CID: 610161, (b) CID: 610186, (c) CID: 91738, and (d) CID: 15074310 into the active site of the *α*-glucosidase, where green represents hydrogen bond; orange represents a salt bridge, attractive charge, and Pi-anion; pink represents Pi-Pi T-shaped, alkyl, and Pi-alkyl; and red represents unfavorable positive–positive.

**Figure 11:**
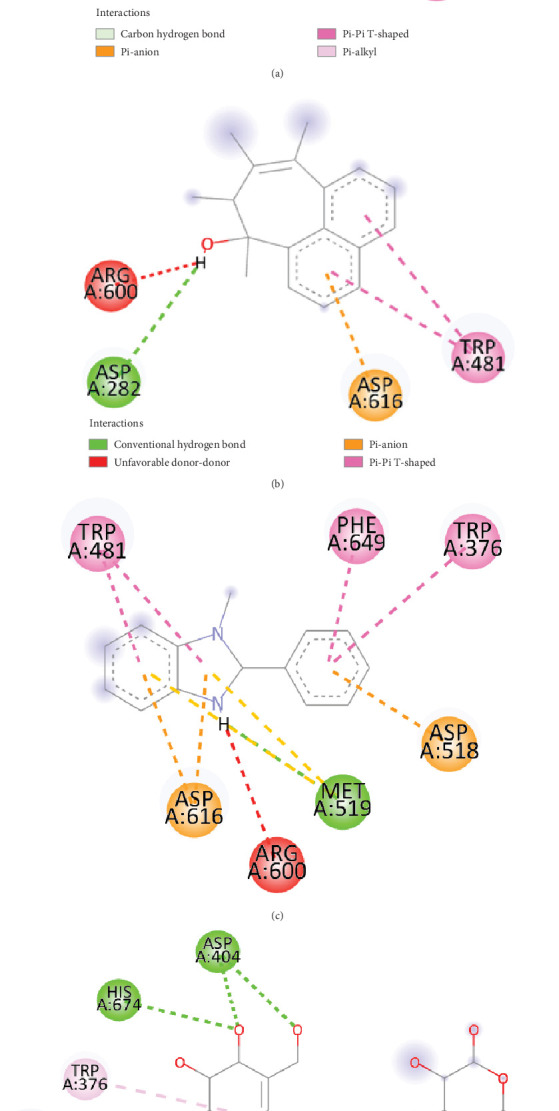
Binding pose and molecular interaction of the mushroom compounds. (a) CID: 610165, (b) CID: 610186, (c) CID: 75807, and (d) acarbose into the active site of the *α*-glucosidase, where green represents hydrogen bond; orange represents a salt bridge, attractive charge, and Pi-anion; pink represents Pi-Pi T-shaped, alkyl, and Pi-alkyl; and red represents unfavorable positive–positive.

Six compounds and one control were analyzed by AutoDock Vina to investigate structure–activity relationships between these compounds and the *α*-glucosidase active site. As described in Table 4, fenugreek sample compounds, 2-ethylacridine (CID_610161) and 7-hydroxy-7,8,9,10-tetramethyl-7,8 dihydrocyclohepta [d,e]naphthalen (CID_610186), have binding affinity −7.1 and −7 kcal/mol that is comparable to control drug acarbose (−7.1 kcal/mol). Additionally, mushroom sample compound gamma-cyano-3-methyl-5,10-dihydrobenzo[f]indolizine (CID_610165), has a binding affinity of −6.9 kcal/mol. Table S2 shows the binding affinity of all poses of all compounds extracted from both fenugreek and mushroom. Interestingly, 7-hydroxy-7,8,9,10-tetramethyl-7,8 dihydrocyclohepta [d,e]naphthalen (CID_610186) compound is common between the two sources. Figures 10 and 11 show the best docking pose and molecular interaction between selected phytocompounds, control drug, and *α*-glucosidase. Moreover, some conventional and nonconventional bonds with different bond lengths were identified between compounds and target protein, and all of the data are shown in Table 5. According to the result, the control drug (acarbose) formed mostly hydrogen bonds with the target protein, whereas selected compounds formed different categories of bonds such as hydrogen bonds, hydrophobic bonds, electrostatic bonds with various types of bonds like conventional hydrogen bonds, Pi-Pi T-shaped, Pi-alkyl, Pi-anion.

## 4. Discussion

Diabetes mellitus is a complex metabolic illness that has significantly impacted human health and quality of life [42]. The global prevalence of diabetes is rapidly increasing, and it can result in both acute and chronic consequences such as retinopathy leading to blindness, nephropathy leading to kidney failure, heart disease, stroke, and amputations. Effective glycemic control requires a well-planned treatment strategy, but no current medication can fully cure the condition, and existing treatments may not work for all patients. Nevertheless, insulin and oral hypoglycemic agents remain the primary pharmaceutical options for managing diabetes mellitus.

In low-income countries, medicinal plants that effectively regulate blood glucose levels with minimal adverse effects are commonly used as alternative therapies for managing diabetes mellitus [43]. Rats with fasting glucose more than 7 mmol/L were considered as diabetic model rats [35], which is similar criteria suggested by ADA/WHO for humans, but the fasting blood glucose of the diabetic model rats used in this study was about 10 mmol/L which is close to other studies [44, 45]. OGTT report of this diabetic model rats showed about 15 mmol/L blood glucose level after 90 min of glucose load, and medicinal plants have long been utilized in traditional medicine systems worldwide for the treatment of diabetes mellitus, owing to their rich biological compounds and known efficacy against the disease. Medicinal herbs with antihyperglycemic properties are becoming more popular because of their fewer adverse effects and are inexpensive [42]. This study investigated medicinal plants like oyster mushrooms and fenugreek for their effects on glucose levels in diabetic model rats. STZ-induced diabetic rats were used in this study, as this model has been widely employed in previous research to screen antidiabetic drugs. Due to its established relevance in such studies, the STZ-induced diabetic rat model was chosen for our investigation [43, 46]. Throughout the study, body weight increased in all five groups of rats, but the MT group exhibited the smallest gain (5.8%). Additionally, after 8 weeks of oral supplement with 5% oyster mushroom and fenugreek, there was a significant reduction in fasting blood glucose levels in STZ-induced diabetic rats. This finding aligns with the results observed in human studies [47]. Besides that, this experimental study also found that serum HDL levels increased significantly (*p* = 0.001), and LDL levels decreased significantly (*p* = 0.0001) in MT diabetic rats compared to DC rats. On the other hand, FT diabetic rats showed no significant changes in HDL value but showed a significant (*p* = 0.0001) decrease in LDL values. The findings of MT rats are very aligned with another published study, although it was done on mice [48]. Our study also looked at total antioxidant status (TAS). The TAS level increased significantly (*p* = 0.01) in diabetic model rats treated with mushrooms, whereas there was no effect on the TAS level in FT diabetic model rats. So, mushroom-contained diet may have a better impact on the TAS level of diabetic individuals than fenugreek-contained diet.

The primary goals of this study were to observe whether the glucose-lowering effects of oyster mushrooms and fenugreek functions through the activation of the AMPK signaling pathway, that is, the insulin-independent pathway, without stimulating pancreatic beta-cells. Interestingly, we found that after 8 weeks of treatment with oyster mushroom and fenugreek, the activated p-AMPK levels in STZ-induced diabetic rats were improved significantly, as shown in band intensity. Additionally, we found that AMPK phosphorylation was reduced in STZ-induced diabetic rats, whereas the MT and FT animals appeared to have enhanced p-AMPK levels. A previous study reported that AMPK activation not only reduces glucose levels but may also help lower the risk of cardiovascular disease by controlling high levels of triglyceride (TG), cholesterol, and free fatty acids [49]. Another study indicated that pharmacological activation of AMPK in insulin-resistant rats enhances the management of hyperglycemia, dyslipidemia, and hypertension. Consequently, identifying the pharmacological compounds that can activate AMPK has gained significant attention as a promising therapeutic target for the treatment of T2D [50]. As a result, one of the main assumptions of this study is that p-AMPK may lower blood glucose levels through the insulin-independent mechanism. Based on previous evidence of the antidiabetic properties of oyster mushrooms and fenugreek, this study is aimed at investigating the effects of these herbs on the activation of proteins associated with the insulin signaling pathway. Specifically, we focused on the activation of p-INSR in STZ-induced diabetes model rats. In this study, the intensity of the p-INSR band intensity seems to be higher in T2D model rats compared to nondiabetic animals. In contrast, this intensity was lower in the muscle tissues of diabetic model rats treated with mushroom and fenugreek since insulin secretion was reduced. We looked at the muscular tissues of GT rats as a positive control and found that it also elevated the p-INSR band intensity. Therefore, mushroom and fenugreek reduced blood glucose—via an insulin-independent mechanism, that is, AMPK pathway [51]. AMPK activation is accomplished via the phosphorylation of its Thr172 residue. Once activated, AMPK can phosphorylate the Akt substrate of 160 kDa (AS160), which directly interacts with the glucose transporter-4 storage vesicles (GSVs) and triggers GLUT-4 trafficking to the plasma membrane [52–55]. Furthermore, according to earlier research, activation of AMPK regulates transcription of the GLUT-4 gene in cultured human skeletal muscle cells [56]. That is why this study is aimed at investigating the effects of oyster mushroom and fenugreek consumption on the expression of the GLUT-4 gene in the muscle tissues of T2D model rats. Our experiment documented a high level of GLUT-4 expression in the tissues of model diabetic rats treated with mushroom powder and fenugreek seed powder, which facilitate glucose absorption through an insulin-independent mechanism. The histological alterations in the Langerhans islets brought on by STZ induction significantly improved in diabetic rats given fenugreek and mushrooms. It has been suggested that the regeneration of pancreatic cells may result from a reduction in oxidative stress, facilitated by plant extracts with antioxidant properties.

All of the phytochemicals obtained from fenugreek and mushrooms through GC-MS analysis were screened to select potent compounds that have good oral bioavailability. To be a bioactive compound, it has to follow the Lipinski rule of five (RO5) [57]. Compounds from both samples (CID_610161, CID_610186, CID_91738, CID_15074310, CID_610165, and CID_75807) fulfilled the conditions of RO5 (shown in Table 3); therefore, these compounds may have good oral bioavailability. Additionally, to understand the comprehensive interaction between selected compounds and *α*-glucosidase, these compounds were evaluated for binding site inhibition at the molecular level. In this study, molecular docking results suggested that these selected bioactive compounds from fenugreek and mushrooms could bind at the same site, the entrance area of the active site of the *α*-glucosidase. *α*-Glucosidase crystal structure–related previous study revealed that the active site of the protein is constructed with Trp 376, Asp 404, Ile 441, Trp 481, Trp 516, Asp 518, Met 519, Arg 600, Trp 613, Asp 616, Phe 649, and His 674 residues [58]. Our selected compounds also bind with these active site residues by various bonds (hydrogen bond, hydrophobic bond, and electrostatic bond), like control drugs. Molecular docking results (the best pose of each compound with *α*-glucosidase residues) are revealed in Figures 10 and 11. Additionally, the list of key residues of *α*-glucosidase active pockets involved in the interaction with selected compounds is summarized in Table 5. Docking results revealed that all compounds were effective at inhibiting *α*-glucosidase activity, but to varying degrees, as demonstrated by binding affinity and the formation of hydrogen, hydrophobic, and electrostatic bonds.

## 5. Conclusion

The findings indicate that both oyster mushrooms and fenugreek exhibit antihyperglycemic properties, demonstrating their effectiveness in improving hyperglycemia and dyslipidemia. Although both mushrooms and fenugreek do not affect insulin secretion or synthesis, our research shows that mushrooms can improve the TAS of diabetes model rats. Furthermore, our research has found that oyster mushrooms and fenugreek do not influence INSR activation, but both of them have a positive impact on AMPK level activation. Another key discovery of our research is that oyster mushrooms and fenugreek can greatly boost GLUT-4 mRNA expression and thereby lower blood glucose levels via an insulin-independent mechanism. Additionally, in silico studies revealed that mushroom and fenugreeks selective phytocompounds might reduce postprandial blood glucose levels by inhibiting *α*-glucosidase activity. As a result, the antihyperglycemic properties of mushrooms and fenugreek may be explained by an insulin-independent glucose absorption mechanism and *α*-glucosidase inhibition. The next target of our research is to use the possible active compounds for in vivo experiments to find out the effective antihyperglycemic compounds for the treatment of diabetes that work the insulin-independent pathway.

### 5.1. Limitations of This Study

The significant limitations of this study are that it did not assess glycated hemoglobin or advanced glycation end-products in diabetic model rats, and the toxicity effect of these herbs was not studied. This study did not conduct an acute test (single dose) of mushroom or fenugreek and also not conduct combined tests of those materials.

## Figures and Tables

**Table 1:** Serum glucose level at 0, 30, 60, and 90 min of oral glucose tolerance test (OGTT).

**Group**	**OGTT (mmol/L)**			
	**0 min**	**30 min**	**60 min**	**90 min**
NDC (*n* = 6)	5.60 ± 0.33^a^	8.26 ± 0.657	8.82 ± 1.68	8.79 ± 1.06
DC (*n* = 6)	11.53 ± 0.9^b^	15.37 ± 4.53	17.58 ± 4.28	18.0 ± 4.79
GT (*n* = 6)	11.30 ± 0.6^b^	17.192 ± 4.41	21.53 ± 6.86	19.89 ± 4.96
MT (*n* = 6)	10.56 ± 1.1^b^	15.06 ± 4.0	17.01 ± 5.25	16.97 ± 4.32
FT (*n* = 6)	10.98 ± 2.1^b^	19.61 ± 2.54	23.30 ± 3.28	22.97 ± 3.48

*Note:* Data presented as mean ± standard deviation (*M* ± SD). Values with different superscripts are significantly different (*p* = 0.0001) in the ANOVA (Bonferroni) test.

Abbreviations: DC, diabetic control; FT, fenugreek treated; GT, glibenclamide treated; MT, mushroom treated; NDC, nondiabetic control.

**Table 2:** Effect of mushroom and fenugreek on serum triglyceride (TG), total cholesterol, HDL cholesterol, and LDL cholesterol levels of Type 2 diabetic model rats.

**Group**	**Triglyceride (mg/dL)**		**Total cholesterol (mg/dL)**		**HDL cholesterol (mg/dL)**		**LDL cholesterol (mg/dL)**	
	**0 day**	**56 days**	**0 day**	**56 days**	**0 day**	**56 days**	**0 day**	**56 days**
NDC	87.81 ± 3	118.01 ± 1 (34.39%)	57.78 ± 7	84.93 ± 3 (46.99%)	21.82 ± 1	16.99 ± 1 (−22.14%)	24.41 ± 9	37.04 ± 11 (51.74%)
DC	113.94 ± 5	146.23 ± 1 (28.34%)	68.42 ± 3	67.34 ± 5 (−1.58%)	23.36±2	18.46 ± 3 (−20.98%)	17.42 ± 7	27.95 ± 6 (60.45%)
GT	161.38 ± 1	158.12 ± 3 (−2.02%)	67.81 ± 2	59.09 ± 6 (−12.86%)	18.49 ± 1	22.05 ± 0.7 (19.25%)	28.59 ± 5	16.79 ± 1 (−41.27%)
MT	168.07 ± 9	174.92 ± 5 (4.08%)	70.03 ± 3	64.77 ± 5 (−7.47%)	17.26 ± 0.4	21.70 ± 1 (25.72%)	29.12 ± 1	19.53 ± 1 (−32.93%)
FT	144.21 ± 1	149.32 ± 9 (3.54%)	66.47 ± 3	79.45 ± 4 (19.53%)	21.11 ± 0.6	22.00 ± 1 (4.22%)	31.57 ± 3	21.26 ± 1 (−32.66%)
Paired *t* -test	*p* values, 0 day vs. 56^th^ day							
NDC	0.0001		0.001		0.010		0.187	
DC	0.005		0.647		0.069		0.145	
GT	0.069		0.043		0.002		0.001	
MT	0.07		0.151		0.001		0.0001	
FT	0.482		0.001		0.260		0.0001	

*Note:* Data presented as mean ± standard deviation (*M* ± SD). Percent values of decrease or increase are presented within parentheses.

Abbreviations: DC, diabetic control; FT, fenugreek treated; GT, glibenclamide treated; MT, mushroom treated; NDC, nondiabetic control.

**Table 3:** List of pharmacokinetic properties includes physicochemical properties, lipophilicity, water solubility, drug likeness, and medicinal chemistry of four selected compounds.

**Properties**		**PubChem ID: 610161**	**PubChem ID: 610186**	**PubChem ID: 91738**	**PubChem ID: 15074310**	**PubChem ID: 610165**	**PubChem ID: 75807**	**PubChem ID: 9811704**
Physiochemical properties	MW (g/mol)	207.27	252.35	326.22	272.22	208.26	208.26	645.6
	Rotatable bonds	1	0	4	4	0	1	13
	H-bond acceptor	1	1	3	6	1	1	19
	H-bond donor	0	1	1	0	0	0	14
	TPSA (Å^2^)	12.89	20.23	50.94	116.36	28.72	17.82	329.01

Lipophilicity	cLogP	4.01	3.49	4.3	3.36	2.41	3.1	−8.82

Water solubility	cLogS	−4.23	−3.99	−4.7	−3.82	−3.16	−3.71	2.57

Pharmacokinetics	GI absorption	High	High	High	High	High	High	Low

Drug likeness	Lipinski	Yes	Yes	Yes	Yes	Yes	Yes	3 violations

Medi. chemistry	Synth. accessibility	Easy	Easy	Easy	Easy	Easy	Easy	Moderate difficult

**Table 4:** List of compound identity, chemical name, 2D structure of selected best six compounds, and acarbose (control) with their binding affinity.

**Source**	**Compounds' PubChem CID**	**Chemical name**	**2D structure**	**Binding affinity (kcal/mol)**
Fenugreek	610161	2-Ethylacridine	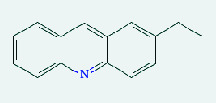	−7.1
	610186	7-Hydroxy-7,8,9,10-tetramethyl-7,8 -dihydrocyclohepta[d,e]naphthalen	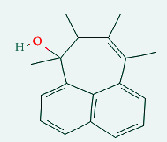	−7
	91738	1-(2,4-Dichlorophenyl)-4,4-dimethyl-2-(1,2,4-triazol-1-yl) pent-1-en-3-ol	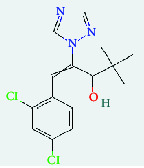	−6.9
	15074310	Diazene, 1-(2,4-dinitrophenyl)-2-phenyl	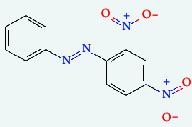	−6.8

Mushroom	610165	Gamma-cyano-3-methyl-5,10-dihydrobenzo[f]indolizine	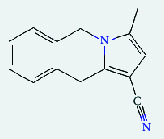	−6.9
	610186	7-Hydroxy-7,8,9,10-tetramethyl-7,8 -dihydrocyclohepta[d,e]naphthalen	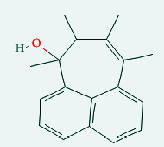	−7
	75807	1-Methyl-2-phenylbenzimidazole	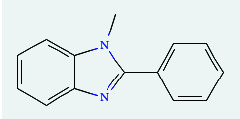	−6.7

Ref. drug	9811704	Acarbose	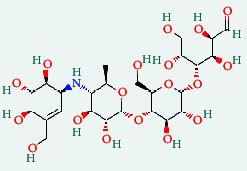	−7.1

**Table 5:** Nonbond interactions between amino acid residues of *α*-glucosidase and our selected six compounds along with acarbose given below.

**PubChem CID**	**Residues name**	**Distance**	**Category**	**Types**
610161	ASP616	2.56542	Hydrogen bond	Conventional hydrogen bond
	ASP518	3.70832	Electrostatic	Pi-anion
	ASP616	3.776	Electrostatic	Pi-anion
	TRP481	4.9588	Hydrophobic	Pi-Pi T-shaped
	PHE649	4.75321	Hydrophobic	Pi-Pi T-shaped
	TRP376	5.49505	Hydrophobic	Pi-alkyl
	TRP481	5.06982	Hydrophobic	Pi-alkyl

610186	ASP282	2.81911	Hydrogen bond	Conventional hydrogen bond
	ASP616	3.91983	Electrostatic	Pi-anion
	TRP481	5.17863	Hydrophobic	Pi-Pi T-shaped
	TRP481	5.49309	Hydrophobic	Pi-Pi T-shaped

91738	ASP616	2.30787	Hydrogen bond	Conventional hydrogen bond
	ASP282	2.38135	Hydrogen bond	Conventional hydrogen bond
	ASP282	3.37051	Hydrogen bond	Carbon hydrogen bond
	LEU678	4.54397	Hydrophobic	Alkyl
	PHE649	5.13381	Hydrophobic	Pi-alkyl

15074310	ASP616	1.94324	Electrostatic	Salt bridge
	ASP616	3.38016	Electrostatic	Attractive charge
	ASP518	5.14113	Electrostatic	Attractive charge
	ARG600	2.35008	Hydrogen bond	Conventional hydrogen bond
	ASP518	3.729	Electrostatic	Pi-anion
	ASP616	3.45016	Electrostatic	Pi-anion
	PHE649	5.10779	Hydrophobic	Pi-Pi stacked

610165	ASP616	3.13766	Hydrogen bond	Carbon hydrogen bond
	ASP518	3.72643	Electrostatic	Pi-anion
	ASP616	3.70532	Electrostatic	Pi-anion
	PHE649	4.74097	Hydrophobic	Pi-Pi T-shaped
	TRP481	5.07596	Hydrophobic	Pi-Pi T-shaped
	TRP481	5.42507	Hydrophobic	Pi-alkyl
	PHE649	5.24249	Hydrophobic	Pi-alkyl

75807	MET519	2.75744	Hydrogen bond	Conventional hydrogen bond
	ASP518	3.60529	Electrostatic	Pi-anion
	ASP616	3.94836	Electrostatic	Pi-anion
	ASP616	4.77196	Electrostatic	Pi-anion
	MET519	4.83151	Other	Pi-sulfur
	MET519	5.70803	Other	Pi-sulfur
	TRP376	5.73244	Hydrophobic	Pi-Pi stacked
	PHE649	5.23146	Hydrophobic	Pi-Pi stacked
	TRP481	5.80941	Hydrophobic	Pi-Pi T-shaped
	TRP481	5.24604	Hydrophobic	Pi-Pi T-shaped
	TRP481	4.81132	Hydrophobic	Pi-Pi T-shaped

9811704	TRP481	3.0316	Hydrogen bond	Conventional hydrogen bond
	TRP481	2.52193	Hydrogen bond	Conventional hydrogen bond
	ASN524	2.6899	Hydrogen bond	Conventional hydrogen bond
	ARG600	2.2193	Hydrogen bond	Conventional hydrogen bond
	ARG600	3.08028	Hydrogen bond	Conventional hydrogen bond
	TRP613	2.54614	Hydrogen bond	Conventional hydrogen bond
	ASN524	2.66374	Hydrogen bond	Conventional hydrogen bond
	ASP616	2.76764	Hydrogen bond	Conventional hydrogen bond
	ASP282	2.09245	Hydrogen bond	Conventional hydrogen bond
	ASP404	2.84749	Hydrogen bond	Conventional hydrogen bond
	ASP518	2.10061	Hydrogen bond	Conventional hydrogen bond
	ASP518	2.3755	Hydrogen bond	Conventional hydrogen bond
	ASP282	3.50104	Hydrogen bond	Carbon hydrogen bond
	ASP404	3.41257	Hydrogen bond	Carbon hydrogen bond

## Data Availability

The datasets used and/or analyzed during the current study are available from the corresponding author upon reasonable request.

## Supporting Information

Supporting Information 1 Table S1: phytochemical composition of fenugreek and mushroom obtained by GC-MS analysis.

Supporting Information 2 Table S2: binding affinity of all poses of all compounds extracted from both fenugreek and mushroom.
