# Supplementary material for: The Intricate Mechanisms of Functional Foods Oyster Mushroom and Fenugreek on Type 2 Diabetic Animal Model
Source: J Diabetes Res. 2024 Dec 9;2024:6209785. doi: 10.1155/jdr/6209785 (PMC11779994; doi:10.1155/jdr/6209785)
Supplement: Supporting Information 1 — Table S1: phytochemical composition of fenugreek and mushroom obtained by GC-MS analysis. [file 6209785.f1.docx]

| **Table S1:** Phytochemical composition of Fenugreek and Mushroom | | | | |
| --- | --- | --- | --- | --- |
| **Alcohol-extracted Fenugreek phytochemicals** | | | | |
| S.L.  No. | Retention  time (RT) | Name of the compounds | Molecular  weight | % Area |
| 1 | 3.53 | Propane, 2-chloro-2-nitro | 123 | 41.38 |
| 2 | 5.44 | Oxime-, methoxy-phenyl- | 151 | 15.48 |
| 3 | 8.53 | 2-propanone, 1,1,3-trichloro | 160 |  |
| 4 | 11.32 | Propane, 1,1-dichloro | 112 | 2.17 |
| 5 | 12.88 | Benzo-1,2,3-triazin-4(3h)-one, 3-amino | 162 | 1.12 |
| 6 | 13.92 | 1,4-pentanediamine | 102 | 1.26 |
| 7 | 15.22 | Chloroacetic acid, heptyl ester | 192 | 1.58 |
| 8 | 17.44 | Carbonic acid, decyl undecyl ester | 356 | 0.56 |
| 9 | 17.58 | 3-n-hexylthiane, s,s-dioxide | 218 | 0.67 |
| 10 | 18.91 | Benzeneethanamine, n [(pentafluorophenyl)methylene]-.beta.,3,4-tris | 563 | 1.10 |
| 11 | 19.79 | Propionic acid, 3-iodo-, octadecyl ester | 452 | 0.49 |
| 12 | 21.31 | 1h-isoindole-1,3(2h)-dione, 2-methyl- | 161 | 0.67 |
| 13 | 22.15 | 2-ethylacridine | 207 | 1.88 |
| 14 | 24.95 | 4-ethylbenzoic acid, 4-hexadecyl ester | 374 | 0.99 |
| 15 | 25.88 | Bupivacaine | 288 | 1.19 |
| 16 | 27.51 | 3,4-dihydroxyphenylglycol | 170 | 1.05 |
| 17 | 28.27 | Heptacosanoic acid, 25-methyl-, methyl ester | 438 | 0.59 |
| 18 | 29.79 | 4-ethylbenzoic acid, 2-methylbutyl ester | 220 | 5.44 |
| 19 | 31.92 | 4-isopropylbenzenethiol, s-methyl | 166 | 2.80 |
| 20 | 33.90 | Butanoyl chloride, 2-chloro | 140 | 1.95 |
| 21 | 35.76 | 2,5-dihydroxybenzoic acid | 154 | 3.16 |
| 22 | 37.76 | 1,1,4,4-tetramethylcyclodecane | 196 | 2.65 |
| **Water-extracted Fenugreek phytochemicals** | | | | |
| S.L  No. | Retention  time (RT) | Name of the compounds | Molecular  weight | % Area |
| 1 | 4.07 | 1,3-oxathiolane, 2-methyl-2-(4-nitrophenyl)- | 225 | 0.83 |
| 2 | 4.37 | Propane, 2-chloro-2-nitro | 123 | 11.44 |
| 3 | 5.92 | 4-ethylbenzoic acid, cyclopentyl ester | 218 | 36.13 |
| 4 | 8.66 | 7-hydroxy-7,8,9,10-tetramethyl-7,8-dihydrocyclohepta[d,e]naphthalen | 252 | 5.15 |
| 5 | 11.31 | 1-(2,4-dichlorophenyl)-4,4-dimethyl-2-(1,2,4-triazol-1-yl)pent-1-en-3-ol | 397 | 0.91 |
| 6 | 12.94 | Hexadecylamine, n-allyl- | 281 | 0.97 |
| 7 | 13.96 | 1,4-pentanediamine | 102 | 0.59 |
| 8 | 15.22 | Anthracene, 9-ethyl-9,10-dihydro-9,10-dimethyl- | 236 | 0.80 |
| 9 | 17.46 | Hexadecane | 226 | 0.26 |
| 10 | 18.92 | Benzeneethanamine, n-[(pentafluorophenyl)methylene]-.beta.,3,4-tris | 563 | 0.52 |
| 11 | 22.15 | 2-ethylacridine | 207 | 1.27 |
| 12 | 24.96 | 4-ethylbenzoic acid, 4-hexadecyl ester | 374 | 0.58 |
| 13 | 27.51 | 4-ethylbenzoic acid, 2-butyl ester | 206 | 0.51 |
| 14 | 29.81 | 4-ethylbenzoic acid, 2-methylbutyl ester | 220 | 2.69 |
| 15 | 31.92 | 4-isopropylbenzenethiol, s-methyl | 166 | 1.00 |
| 16 | 33.91 | Butanoyl chloride, 2-chloro | 140 | 0.80 |
| 17 | 35.77 | 4-chloro-2-isothiocyanato-1-phenoxybenzene | 261 | 20.05 |
| 18 | 37.77 | Diazene, 1-(2,4-dinitrophenyl)-2-phenyl | 272 | 9.72 |
| **Alcohol-extracted Mushroom phytochemicals** | | | | |
| S.L  No. | Retention  time (RT) | Name of the compounds | Molecular  weight | % Area |
| 1 | 3.26 | Thietane, 2,4-dimethyl- | 102 | 0.95 |
| 2 | 3.63 | Propane, 2-chloro-2-nitro | 123 | 29.13 |
| 3 | 5.45 | Oxime-, methoxy-phenyl- | 151 | 15.93 |
| 4 | 7.49 | Trifluoroacetonitrile | 95 | 1.16 |
| 5 | 8.50 | 7-hydroxy-7,8,9,10-tetramethyl-7,8-dihydrocyclohepta[d,e]naphthalen | 252 | 2.83 |
| 6 | 9.54 | Boronic acid, ethyl-, bis(2-mercaptoethyl ester) | 194 | 2.20 |
| 7 | 11.31 | 1-(2,4-dichlorophenyl)-4,4-dimethyl-2-(1,2,4-triazol-1-yl)pent-1-en-3-ol | 326 | 1.81 |
| 8 | 15.23 | 2,6-dihydroxyacetophenone | 152 | 1.63 |
| 9 | 17.45 | Dodecane, 1-fluoro- | 188 | 0.51 |
| 10 | 18.90 | Benzeneethanamine, n-[(pentafluorophenyl)methylene]-.beta.,3,4-tris | 563 | 0.83 |
| 11 | 19.81 | Hentriacontane | 436 | 0.50 |
| 12 | 21.31 | 1h-isoindole-1,3(2h)-dione, 2-methyl- | 161 | 1.46 |
| 13 | 22.15 | .Gamma.-cyano-3-methyl-5,10-dihydrobenzo[f]indolizine | 208 | 2.02 |
| 14 | 23.05 | .Alpha.-d-glucopyranosiduronamide, methyl 2,3-di-o-methyl | 235 | 1.00 |
| 15 | 24.55 | 3-buten-2-ol, benzyldimethylsilyl ether | 220 | 0.94 |
| 16 | 24.95 | 1,4-dioxane-2,5-diol | 120 | 0.64 |
| 17 | 27.49 | 3,4-dihydroxyphenylglycol | 170 | 0.90 |
| 18 | 28.24 | Tetradecanoic acid, 10,13-dimethyl-, methyl ester | 270 | 1.55 |
| 19 | 29.81 | Cyclopentaneundecanoic acid, methyl ester | 268 | 2.67 |
| 20 | 31.91 | Undecanoic acid, 10-methyl-, methyl ester | 214 | 3.02 |
| 21 | 33.91 | Methyl 8-methyl-nonanoate | 186 | 1.62 |
| 22 | 35.76 | Heptadecanoic acid, 16-methyl-, methyl ester | 298 | 9.34 |
| 23 | 37.77 | Methyl 18-fluoro-octadecanoate | 316 | 3.73 |
| **Water-extracted Mushroom phytochemicals** | | | | |
| S.L  No. | Retention  time (RT) | Name of the compounds | Molecular  weight | % Area |
| 1 | 3.59 | Propane, 1-chloro-2-nitro | 123 | 44.61 |
| 2 | 3.63 | Propane, 2-chloro-2-nitro- | 123 | 0.10 |
| 3 | 5.44 | Oxime-, methoxy-phenyl- | 151 | 18.38 |
| 4 | 7.51 | 9 cyclopentanemethanol, .alpha.-(1-methylethyl)-2-nitro-, [1.alpha.(s*),2. | 187 | 0.63 |
| 5 | 8.52 | 7-hydroxy-7,8,9,10-tetramethyl-7,8-dihydrocyclohepta[d,e]naphthalen | 252 | 1.00 |
| 6 | 10.12 | Benzeneacetaldehyde | 120 | 0.66 |
| 7 | 11.31 | Undetermined |  |  |
| 8 | 15.23 | Undetermined |  |  |
| 9 | 17.59 | 3-n-hexylthiane, s,s-dioxide | 218 | 0.82 |
| 10 | 18.92 | Benzeneethanamine, n-[(pentafluorophenyl)methylene]-.beta.,3,4-tris | 563 | 1.12 |
| 11 | 21.31 | 1h-isoindole-1,3(2h)-dione, 2-methyl- | 161 | 0.88 |
| 12 | 22.17 | 1-methyl-2-phenylbenzimidazole | 208 | 1.56 |
| 13 | 23.14 | 1h-pyrrole-3,4-dicarbonitrile, 2,5-dihydro-2,5-dioxo- | 147 | 0.43 |
| 14 | 24.96 | 1,4-dioxane-2,5-diol | 120 | 1.04 |
| 15 | 27.50 | 3,4-dihydroxyphenylglycol | 170 | 1.30 |
| 16 | 29.80 | Tetradecanoic acid, 10,13-dimethyl-, methyl ester | 270 | 2.79 |
| 17 | 31.92 | Cyclopentaneundecanoic acid, methyl ester | 268 | 2.51 |
| 18 | 33.90 | Undecanoic acid, 10-methyl-, methyl ester | 214 | 2.89 |
| 19 | 35.77 | Methyl 8-methyl-nonanoate | 186 | 3.07 |
| 20 | 37.77 | Heptadecanoic acid, 16-methyl-, methyl ester | 298 | 3.41 |
